# Regulatory Effects of CsrA in Vibrio cholerae

**DOI:** 10.1128/mBio.03380-20

**Published:** 2021-02-02

**Authors:** Heidi A. Butz, Alexandra R. Mey, Ashley L. Ciosek, Alexander A. Crofts, Bryan W. Davies, Shelley M. Payne

**Affiliations:** a Department of Molecular Biosciences and LaMontagne Center for Infectious Diseases, The University of Texas at Austin, Austin, Texas, USA; University of Würzburg

**Keywords:** *Vibrio cholerae*, CsrA, regulon, AphA

## Abstract

CsrA is a posttranscriptional global regulator in Vibrio cholerae. Although CsrA is critical for V. cholerae survival within the mammalian host, the regulatory targets of CsrA remain mostly unknown. To identify pathways controlled by CsrA, RNA-seq transcriptome analysis was carried out by comparing the wild type and the *csrA* mutant grown to early exponential, mid-exponential, and stationary phases of growth. This enabled us to identify the global effects of CsrA-mediated regulation throughout the V. cholerae growth cycle. We found that CsrA regulates 22% of the V. cholerae transcriptome, with significant regulation within the gene ontology (GO) processes that involve amino acid transport and metabolism, central carbon metabolism, lipid metabolism, iron uptake, and flagellum-dependent motility. Through CsrA-RNA coimmunoprecipitation experiments, we found that CsrA binds to multiple mRNAs that encode regulatory proteins. These include transcripts encoding the major sigma factors RpoS and RpoE, which may explain how CsrA regulation affects such a large proportion of the V. cholerae transcriptome. Other direct targets include *flrC*, encoding a central regulator in flagellar gene expression, and *aphA*, encoding the virulence gene transcription factor AphA. We found that CsrA binds to the *aphA* mRNA both *in vivo* and *in vitro*, and CsrA significantly increases AphA protein synthesis. The increase in AphA was due to increased translation, not transcription, in the presence of CsrA, consistent with CsrA binding to the *aphA* transcript and enhancing its translation. CsrA is required for the virulence of V. cholerae and this study illustrates the central role of CsrA in virulence gene regulation.

## Abstract (precis)

Vibrio cholerae, a Gram-negative bacterium, is a natural inhabitant of the aqueous environment. However, once ingested, this bacterium can colonize the human host and cause the disease cholera.

## INTRODUCTION

Vibrio cholerae is a natural inhabitant of the aquatic environment; however, if ingested, toxigenic V. cholerae can colonize the human small intestine and elicit the deadly human disease cholera. To colonize, the bacterium must overcome stomach acidity, resist the antimicrobial agent bile, penetrate the mucosal barrier, and attach to the epithelial surface.

Bile is found within the lumen of the small intestine. The primary function of bile is to emulsify and solubilize lipids for digestion. Because the bacterial membrane is primarily composed of lipids, bile can cause lysis of the bacterium by disrupting cytoplasmic membrane integrity. V. cholerae has evolved many mechanisms to overcome the toxic effects of bile, one of which includes exchanging a bile-permissive outer membrane porin (OMP) OmpT for a bile-nonpermissive porin OmpU (1). The bacteria that survive transit through the lumen must then penetrate through the mucosal barrier that is lining the epithelial surface. The mucosal lining is composed of heavily glycosylated proteins that oligomerize to form a viscous layer. This layer is ∼70× the length of a V. cholerae cell (2); thus, motility is hypothesized to be required for V. cholerae to penetrate the mucosal barrier (3). When the bacteria reach the epithelial surface, they replicate to form microcolonies. Attachment and microcolony formation require the toxin coregulated pilus, TCP (4). The bacteria release cholera toxin (CTX), which is endocytosed by the host epithelial cells and results in the efflux of ions as well as water molecules. This manifests as the major clinical symptom, rice water stool, of a V. cholerae infection, and aids in the dissemination of the bacteria back into the aqueous environment.

Having both an aquatic and host-associated lifestyle requires the regulation of a large number of cellular processes to ensure optimal expression in each environment. An orchestrated response enables the bacterium to adapt to the host environment, promoting colonization and virulence factor production. Global regulatory proteins act in response to environmental cues and are crucial to achieving a rapid, broad, and coordinated change in expression of a large number of downstream target genes. One such global regulator in V. cholerae is the RNA-binding protein CsrA. CsrA was first identified in V. cholerae for its role in the quorum sensing (QS) pathway (5). QS is a cell-to-cell communication system that enables the bacterial population to orchestrate either low-cell-density or high-cell-density behaviors, such as toxin production and biofilm formation, respectively (6). Lenz et al. (5) demonstrated that a mutation in *csrA* resulted in the inability to regulate gene expression in a QS-dependent manner, suggesting the QS signal transduction pathway requires CsrA. Additionally, we have shown that extracellular cues affect CsrA-mediated regulation of ToxR, a master regulator of virulence gene expression. Through genetic analysis, we determined that ToxR levels increase in response to nutrient supplementation, such as the addition of the four amino acids asparagine, arginine, glutamate, and serine (NRES), and that this increase in ToxR is dependent on CsrA (7). Further, we demonstrated CsrA is required for pathogenesis in the infant mouse model (7). Taken together, these data demonstrate that CsrA-mediated regulation is required to elicit virulence gene expression in response to extracellular signals, and that CsrA plays an integral role in the bacterium’s ability to successfully colonize and cause disease in the host. However, as no direct targets of CsrA have been identified in these pathways, the exact mechanism of CsrA-dependent regulation on both QS and ToxR is incompletely understood. The broader regulatory effects of this global regulator also remain unknown in V. cholerae, even though in other bacterial pathogens it has been demonstrated to regulate the expression of hundreds of transcripts (8–10, 69, 70).

In Escherichia coli, where CsrA has been more extensively studied, it has been shown that CsrA often regulates its target RNAs by binding to the 5′ untranslated region (5′ UTR) to either positively or negatively affect transcript stability (11), transcript elongation (12), and/or the efficiency of translation (13, 14). It has been demonstrated in E. coli that CsrA regulates processes that, if conserved, would be critical for the ability of V. cholerae to colonize the host; for example, CsrA-mediated regulation of motility. In E. coli, CsrA positively regulates motility by directly binding to the *flhDC* transcript, which encodes the flagellar master regulator, to prevent RNaseE-mediated degradation, thus increasing its stability (15, 16).

One mRNA known to be a direct target of CsrA in V. cholerae is *varA* (17). We demonstrated that CsrA directly binds to the *varA* mRNA and that CsrA positively regulates VarA protein production. VarA is a transcriptional activator that is homologous to E. coli UvrY (5). VarA induces the expression of three small RNAs (sRNAs) CsrB, CsrC, and CsrD (Csr sRNAs). In E. coli, the gene named *csrD* encodes a protein that controls the degradation of CsrA (18), and MshH plays this role in V. cholerae (19). The three Csr sRNAs contain multiple CsrA-binding sites, characterized by GGA motifs located in the loop region of stem-loop structures in the RNA. When expressed, these sRNAs act to sequester CsrA, thus preventing CsrA from interacting with its target mRNAs. Therefore, CsrA regulation uses a feedback loop, whereby CsrA positively induces the expression of its antagonistic sRNAs. It also indicates that the availability of CsrA, or activity of CsrA, is tightly regulated within the cell.

In this study, we show that CsrA in V. cholerae is a global regulator that affects a large number of gene products. Direct targets for CsrA include mRNAs for regulators that in turn control expression of genes encoding a number of pathways, including those responsible for motility, rugosity, and virulence.

## RESULTS

To better understand the regulatory role of CsrA in V. cholerae, we used RNA-seq transcriptome sequencing to compare the transcriptome of a *csrA* mutant strain to that of its wild-type parental strain, N16961. It should be noted that N16961 does not have an intact quorum sensing (QS) system, and this could lead to differences in the CsrA regulon compared with a QS+ strain. CsrA is essential for the growth of V. cholerae, and therefore a complete deletion of the *csrA* gene is not possible. We have isolated a *csrA* point mutant, N*csrA*.R6H, which has wild-type growth (7) but does not exhibit the CsrA-dependent increase in ToxR levels in response to NRES, indicating that the CsrA.R6H protein is less functional than wild-type CsrA. Significantly, this mutant is avirulent in the infant mouse model of V. cholerae infection (7). The N*csrA*.R6H mutant and the wild-type strain were grown in defined medium in the presence of NRES, which we have shown previously to increase the level of active CsrA (7, 17). To give a more complete picture of the role of CsrA in regulating gene expression at different stages of V. cholerae growth, RNA was harvested from wild type and the *csrA* mutant grown to early exponential, mid-exponential, and stationary phase. Because the N*csrA.*R6H mutant grows as well as its wild-type parental strain in defined medium supplemented with NRES (7), the observed differences in gene expression between the two strains are not due to differences in growth rate or yield.

### CsrA is a global regulator in V. cholerae.

**FIG 1:**
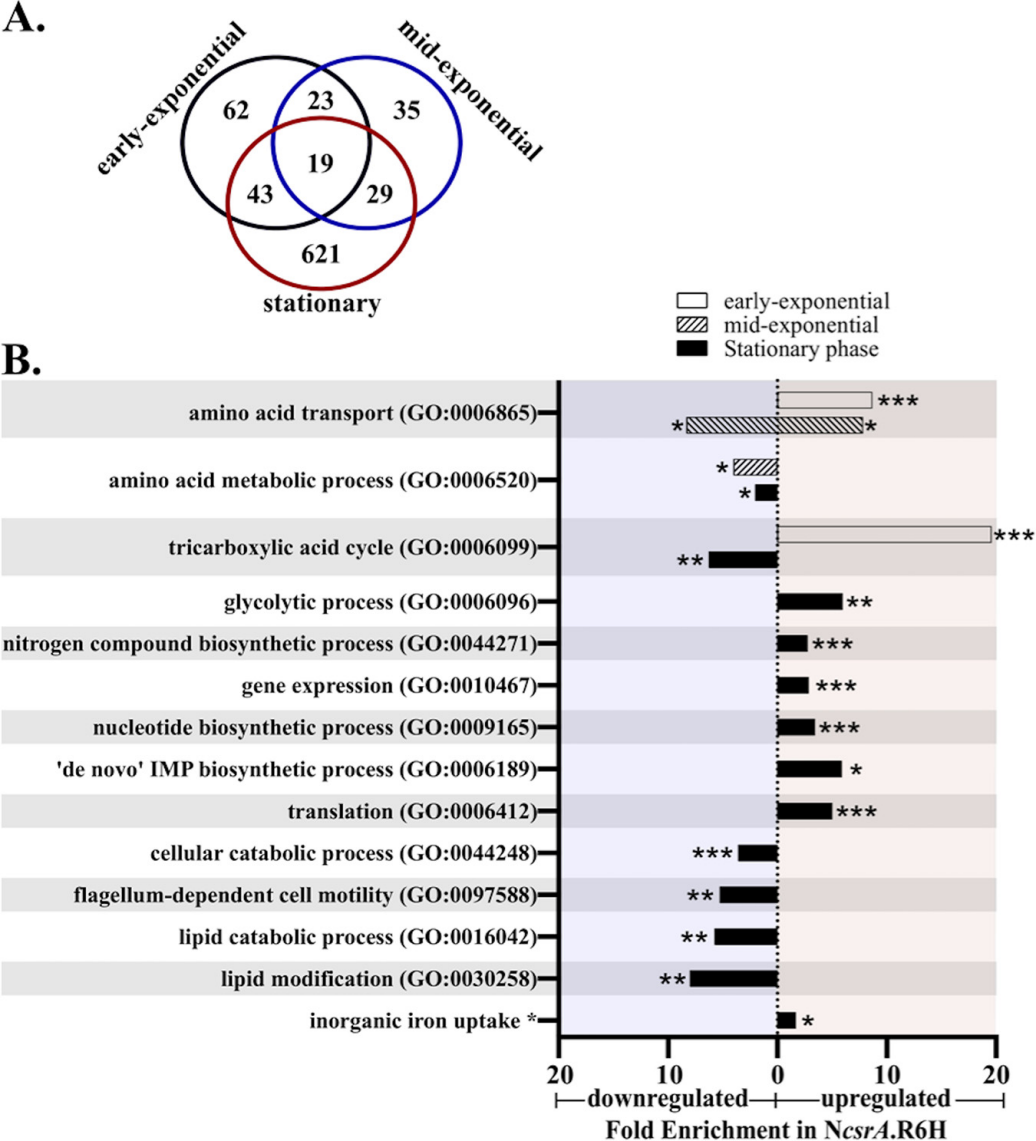
Overview of CsrA regulation in V. cholerae. (A) Venn diagram enumerating all the genes that displayed a greater than 2-fold difference in gene expression and had an FDR *P* value of <0.05 in N*csrA*.R6H compared to N16961. (B) GO ontology analysis performed via PANTHER. The genes included in this analysis had a greater than 2-fold change in expression values. The bars that extend to the left of zero (blue shading) have an overrepresentation of downregulated genes, whereas the bars that extend to the right of zero (orange shading) have an overrepresentation of upregulated genes in the *csrA* mutant compared to the wild type at the indicated growth phase. Fold enrichment, as determined by PANTHER analysis, is the comparison of the regulation of genes in that category to the basal regulation for all genes in comparing the mutant to the wild type RNA-seq data. The Fisher’s exact test was applied to determine significantly regulated categories (FDR *P* values: *, <0.05; **, <0.01; ***, <0.001).

In total, 832 genes were significantly differentially expressed in the N*csrA*.R6H strain compared to the wild type (Fig. 1A). Only genes exhibiting a greater than 2-fold change in expression between the *csrA* mutant and the wild-type strain, with a false discovery rate (FDR) *P* value of less than 0.05, were included in the analysis (Table S1 in the supplemental material). CsrA has been shown to play a major role in regulating stationary-phase gene expression in other bacteria (8, 9, 20) and, not surprisingly, the majority of differentially regulated genes were observed at stationary phase. A total of 712 stationary phase CsrA-regulated genes were identified and, of these, 621 were regulated exclusively in stationary phase, showing that CsrA in V. cholerae, like in E. coli, is a major stationary phase regulator. Each growth phase was associated with the unique expression pattern of at least 35 genes. Additionally, 19 genes were found to be significantly differentially regulated at all phases of growth (Table S1). The genes regulated in all growth phases included the *csrB* and *csrD* genes, 4 transport genes, 5 metabolism genes, the *hlyA* gene encoding hemolysin, and 7 genes encoding proteins with uncharacterized or hypothetical functions.

10.1128/mBio.03380-20.2 TABLE S1 Comparison of gene expression between the wild type and *csrA* mutant at various growth phases. To compare gene expression, NcsrA.R6H and N16961 were grown in minimal medium supplemented with NRES, and RNA samples were harvested at early exponential, mid-exponential, and stationary phase. The Baggerly’s test was used to determine significance and the weighted proportions fold change from 3 biological replicates, comparing the *csrA* mutant to the wild type. Download TABLE S1, XLSX file, 0.4 MB. Copyright © 2021 Butz et al. 2021 Butz et al. This content is distributed under the terms of the Creative Commons Attribution 4.0 International license.

Gene Ontology (GO) groupings were used to characterize the global effects of CsrA regulation on cellular processes (Fig. 1B) (Table S2). The PANTHER (Protein Analysis Through Evolutionary Relationships) bioinformatics web server (http://www.pantherdb.org) (21, 22) was used to determine GO groupings that are overrepresented among significantly regulated genes in the *csrA* mutant compared with the wild-type strain within the three different growth phases. Based on the number of genes in a group, the analysis determined whether the number of genes that are regulated by CsrA is significantly more or less than would be expected by chance. The ratio of observed to expected genes is expressed as fold enrichment (Fig. 1B). From this GO analysis, we found that CsrA affects the expression of multiple categories of processes, and some of the GO groupings are regulated in a growth phase-dependent manner. For example, at early exponential phase, there was a 19.8-fold enrichment of significantly more highly expressed genes in the tricarboxylic acid (TCA) cycle GO group in the *csrA* mutant than in the wild type. Genes in this GO group were also significantly overrepresented in stationary phase but, in contrast to early exponential phase, these genes were expressed at a lower level in the mutant compared to wild type in stationary phase. Genes in the glycolytic process GO group were also overrepresented at stationary phase, but these genes were more highly expressed in the *csrA* mutant than in the wild type. This pattern of expression is consistent with CsrA repressing the TCA cycle early in the growth cycle but allowing TCA cycle genes to be expressed in stationary phase, while at the same time repressing glycolysis in stationary phase. No significant enrichment of glycolysis genes was observed in the *csrA* mutant at either early- or mid-exponential phase.

10.1128/mBio.03380-20.3 TABLE S2 Pathways or processes (Gene Ontologies) that are overrepresented in the *csrA* mutant (R6H) compared to wild-type V. cholerae. The expression data were analyzed by the PANTHER overrepresentation test (released 11 July 2019) to determine which pathways were overrepresented in the mutant. Download TABLE S2, XLSX file, 0.1 MB. Copyright © 2021 Butz et al. 2021 Butz et al. This content is distributed under the terms of the Creative Commons Attribution 4.0 International license.

Nucleotide biosynthesis processes were overrepresented at stationary phase. We observed upregulation of genes of the nucleotide biosynthetic process (3.5-fold overrepresentation) and the *de novo* inosine monophosphate (IMP) biosynthetic process GO groups in the *csrA* mutant at stationary phase. Nitrogen compound biosynthesis was similarly expressed at a higher level in the *csrA* mutant at stationary phase. These observations point to an overall repressive effect of CsrA on nucleotide biosynthesis during stationary-phase growth. CsrA may also act to repress gene expression and protein translation generally in stationary phase, as these two GO groups were both significantly overrepresented in stationary phase, showing a relatively higher expression in the *csrA* mutant. At the same time, cellular catabolic processes, including lipid catabolism, were expressed at lower levels in the mutant. These results point to a role of CsrA in decreasing gene expression and *de novo* protein biosynthesis, while increasing catabolic processes, in response to nutrient depletion in stationary phase. We found that genes encoding several inorganic ferric and ferrous iron uptake systems, including FbpABC (VC0608 to VC0610), FeoA (VC2078), and VctPDGC (VCA0227- to VCA0230) (reviewed in reference 23), were upregulated in the *csrA* mutant in stationary phase, while some genes encoding iron-storage, iron-binding, and iron-containing proteins, such as ferritin (VC0078), ferredoxin (VC0311), and iron-sulfur cluster-containing proteins (VC1512, VC2088, and VCA0985), were repressed. This suggests that CsrA may be critical for restricting the influx of free, unchelated iron during stationary phase growth, while sequestering any excess intracellular iron.

Another significantly overrepresented group in the GO analysis was flagellum-dependent cell motility. Genes whose expression was relatively lower in the *csrA* mutant at stationary phase were overrepresented in this category, suggesting that CsrA is an activator of motility. Of the 25 genes included in this category, 11 were regulated in response to CsrA, suggesting that CsrA affects flagellar gene expression early in the cascade or at multiple points in the flagellar gene expression hierarchy.

Among other notable targets of CsrA regulation observed in the RNA-seq analysis were several sigma factor genes, including *rpoS* (sigma-38, down 7-fold in the *csrA* mutant), *rpoN* (sigma-54, down 2.1-fold in the *csrA* mutant), *rpoE* (sigma-24, down 2-fold in the *csrA* mutant), and *rpoD* (sigma-70, up 2.6-fold in the *csrA* mutant). This pattern of sigma factor regulation suggests a larger picture involving CsrA-mediated upregulation of the stationary-phase stress response (*rpoS*, *rpoE*), with an overall dampening of gene expression through downregulation of the housekeeping sigma factor (*rpoD*). RpoS and RpoN are known to control many processes linked to the virulence of V. cholerae, including motility, quorum sensing, and biofilm formation, and their positive regulation by CsrA further highlight the importance of CsrA for the pathogenesis of V. cholerae. Additional CsrA-regulated genes with known or predicted roles in virulence identified in the RNA-seq analysis include *varA* (VC1213), which we have demonstrated previously to be a direct target of CsrA regulation (17), *hlyA*, encoding the major V. cholerae hemolysin (24), *rstA* and *rstB*, encoded on the CTX-phage (25), and *aphA* (VC2647), which is discussed in detail below.

### Expression of the Csr sRNAs changes over time.

**FIG 2:**
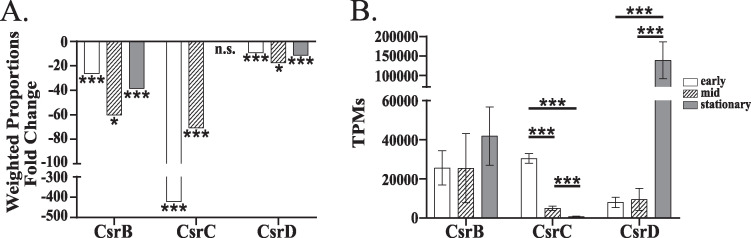
Expression of the Csr sRNAs change over time. (A) The expression of the Csr sRNAs, CsrB, CsrC, and CsrD, in the *csrA* mutant N*csrA*.R6H compared to the wild-type strain N16961. (B) The transcripts per million (TPM) values for each of the Csr sRNAs at different growth phases in N16961. The Baggerley’s method was used to determine statistical significance (FDR *P* values: *, <0.05; ***, <0.001; n.s., not significantly different). Error bars indicate standard deviations.

In our previous work, we demonstrated an autoregulatory feedback loop in which CsrA positively regulates the expression of the three Csr sRNAs (CsrB, CsrC, and CsrD) through its posttranscriptional effects on the expression of *varA*, encoding an activator of transcription of all three Csr sRNAs (17). As the Csr sRNAs function to antagonize the activity of CsrA, this feedback loop likely prevents drastic oscillations of CsrA activity. In support of our original observations, the expression of each of the Csr sRNAs was significantly reduced in the *csrA* mutant (Fig. 2A); however, the extent to which each sRNA level decreased varied in the *csrA* mutant. CsrC showed the most dramatic reduction in response to the loss of CsrA, particularly in early and mid-exponential phase, whereas CsrD was the least affected, despite being the most abundant in the wild type at stationary phase (Fig. 2B). This could suggest intrinsic differences between the sRNAs in their expression and dependence upon *csrA*. Our previous work indicated that the Csr sRNAs are not completely functionally redundant (17), leading us to hypothesize that the expression of the Csr sRNAs may vary in response to different conditions, including growth phase. We examined the expression of the Csr sRNAs in the wild-type strain at each growth phase. We found that the level of CsrB did not significantly differ from one growth phase to the next (Fig. 2B). CsrC expression was highest at early exponential phase, and then decreased over time and had the lowest abundance of the three sRNAs at mid-exponential and stationary phase. In stationary phase, the expression of CsrC was quite low, representing a 41-fold decrease compared to the level in early exponential phase. The levels of CsrD did not change from early to mid-exponential phase, but did increase substantially from mid-exponential to stationary phase. Notably, at stationary phase, CsrD was the most highly expressed RNA in the entire wild type transcriptome and accounted for nearly 10% of the total reads, with 93,842.2 transcripts out of a total of 1,000,000 transcripts. The differential expression of the Csr sRNAs supports the hypothesis that the Csr sRNAs have intrinsically different properties, and that this system is finely tuned to regulate the activity level of CsrA in response to changing environments.

### CsrA binds directly to multiple transcripts encoding regulatory proteins.

**FIG 3:**
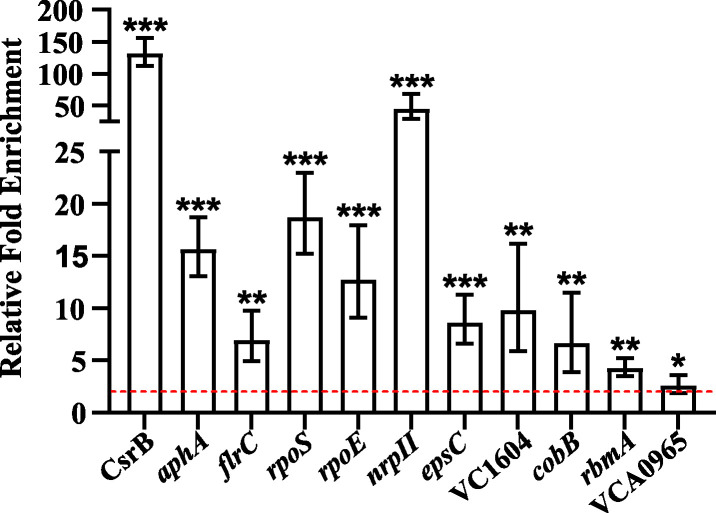
Coimmunoprecipitation of selected mRNA targets with CsrA. CsrA-V5 expression was induced with arabinose at mid-exponential phase. Enrichment was determined via RT-qPCR by determining the relative amount of the target RNA in the immunoprecipitated sample compared to the amount in the initial sample. Dashed red line indicates the cutoff for 2-fold enrichment. The *P* values were determined by an unpaired, two-tailed Student’s *t* test from the Δ*C_T_* values (*P* values: *, <0.05; **, <0.01; ***, <0.001). Error bars indicate standard deviations. Gene VC numbers are *aphA* (VC2647), *flrC* (VC2135), *rpoS* (VC0534), *rpoE* (VC2467), *nprII* (VC2239), *espC* (VC2734), *cobB* (VC1509), and *rbmA* (VC0928).VCA0965 is a functional cyclic di-GMP synthase (30) and VC1604 is annotated as a response regulator.

Because CsrA had such a substantial effect on the V. cholerae transcriptome, we hypothesized that CsrA might be directly regulating multiple regulatory proteins. To test this model, we performed a directed RNA-CsrA coimmunoprecipitation (co-IP) (Fig. 3) using CsrA-overproducing cells grown to mid-exponential phase, and real-time quantitative PCR (RT-qPCR) to determine relative enrichment of specific RNA targets. The targets chosen were mRNAs identified as CsrA-regulated in the RNA-seq analysis and encode known or putative regulatory proteins. The amount of the RNA target was quantified relative to an internal standard in the RNA-CsrA coimmunoprecipitated sample compared with the initial RNA population. As a positive control for the enrichment of direct CsrA targets, CsrB was included in the panel of RNAs investigated. CsrB was enriched over 100-fold in the immunoprecipitated sample, indicating this method can detect direct targets of CsrA. Targets less abundant than CsrB can also be detected through co-IP analysis; our previous work demonstrated that *varA* mRNA, described above as encoding the transcriptional activator of Csr sRNA expression, was enriched 5-fold in the CsrA-coimmunoprecipitated RNA pool (17). We found that the selected mRNAs that encode regulatory proteins coimmunoprecipitated with CsrA. For example, transcripts encoding the sigma factors RpoS and RpoE, the flagellar regulator FlrC, and the QS master regulator AphA were significantly enriched with CsrA. This finding suggests that CsrA may exert large regulatory effects on the V. cholerae transcriptome by controlling the expression of other regulators. Consistent with our observation that CsrA is required for virulence within the host, we identified multiple CsrA-interacting mRNAs associated with pathogenesis, including the following: *aphA* (26); *flrC* (27); *epsC*, which encodes a component of the type two secretion system that is responsible for the secretion of the cholera toxin (28); *cobB*, which encodes an NAD+ dependent deacetylase (29); and VCA0965, encoding a functional cyclic di-GMP synthase (30). Selected regulators are discussed in more detail below.

### CsrA regulates the expression of the stationary phase regulator RpoS.

A large proportion of the CsrA-regulated genes identified in the RNA-seq analysis were exclusive to stationary-phase cells, suggesting that CsrA may be a major regulator of stationary phase gene expression. Expression of *rpoS*, which encodes the stationary phase sigma factor, sigma-38, was downregulated ∼7-fold at stationary phase in the *csrA* mutant (Table S1), suggesting CsrA is critical for induction of *rpoS* in stationary phase. In addition, we found that *rpoS* mRNA coimmunoprecipitated with CsrA (Fig. 3), consistent with *rpoS* being a direct target of CsrA regulation. This result suggests that at least part of the observed regulation of stationary phase gene expression by CsrA may occur via RpoS. To investigate this idea further, we determined whether there was an overlap between the CsrA and RpoS regulons by comparing genes regulated by RpoS at stationary phase, identified by Nielsen et al. (31), to genes regulated by CsrA at stationary phase. It is important to note that these two RNA-seq analyses were performed in different media, which may affect the expression of some of the genes. We found that, at stationary phase, 195 genes exhibited regulation by both CsrA and RpoS. Interestingly, of these genes, 63% are regulated in a similar way by CsrA and RpoS, exhibiting either upregulation or downregulation by both regulators. Taken together, these data suggest that regulation of stationary phase gene expression by CsrA occurs, at least in part, through its control of RpoS levels.

### CsrA positively regulates motility in V. cholerae.

**FIG 4:**
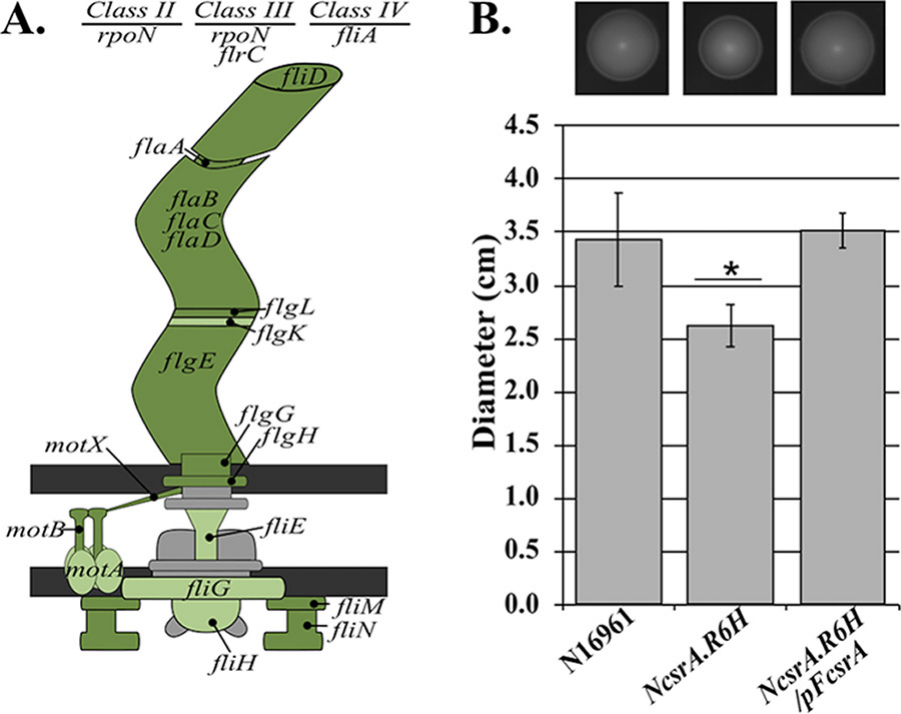
CsrA positively regulates motility in V. cholerae. (A) A reconstruction of the flagellum, as well as the hierarchal regulatory genes, in V. cholerae, where the colored proteins represent genes from the RNA-seq analysis that were significantly differentially regulated in N*csrA*.R6H compared to the wild type (N16961). The genes that were significantly downregulated and displayed a greater than 2-fold change (Baggerley’s test, FDR *P* value of <0.05) in expression in the *csrA* mutant compared with the wild-type strain are shown in dark green, while genes that were significantly downregulated but did not meet the 2-fold change threshold, are shown in light green. (B) The ability of N16961, N*csrA*.R6H, and N*csrA*.R6H/pF*csrA* to swim through semisolid minimal agar supplemented with NRES was assessed. The bars represent the mean of five biological replicates, the error bars are the standard deviations, and the *P* value was calculated using one-way ANOVA (*P* values: *, <0.05) comparing N*csrA*.R6H to both the wild type and the complemented mutant. Error bars indicate standard deviations. The photos are from one experiment and are representative of the zone of growth produced by each strain. Gene VC numbers are *motA* (VC0892); *motB* (VC0893); *fliA* (VC2066); *fliN* (VC2125); *fliM* (VC2126); *fliH* (VC2131); *fliG* (VC2132); *fliE* (VC2134); *flrC* (VC2135); *flrA* (VC2137); f*liD* (VC2140); *flaB* (VC2142); *flaD* (VC2143); *flaC* (VC2187); *flaA* (VC2188); *flgL* (VC2190), *flgK* (VC2191); *flgH* (VC2194); *flgG* (VC2195); *flgE* (VC2200); *rpoN* (VC2529); and *motX* (VC2601).

In our RNA-seq analysis, we found that over half of all annotated flagellum-dependent motility genes had significantly lower expression in the *csrA* mutant relative to the wild-type strain at stationary phase. This difference suggests a major role for CsrA in the regulation of flagellum-dependent motility in stationary phase. Figure 4A shows the flagellar proteins encoded by the genes that were significantly regulated in the RNA-seq analysis. In addition to flagellar structural genes, we found that several regulatory genes involved in flagellar gene expression were differentially expressed in the *csrA* mutant, including *rpoN*, *rpoS*, and *flrC*, suggesting the effects of CsrA on motility gene expression could be due to CsrA-mediated regulation of these central regulators. In support of this, we found that CsrA bound to both *rpoS* and *flrC* mRNA *in vivo*, as determined by the CsrA-RNA coimmunoprecipitation experiments (Fig. 3).

To determine whether the observed regulation of flagellar gene expression by CsrA is relevant for motility in V. cholerae, the ability of the *csrA* mutant to swim through a semisolid agar medium was assessed. The *csrA* mutant strain displayed a reduced zone of spread compared to the wild type, indicating the *csrA* mutant is less motile than the wild type (Fig. 4B). The motility defect was restored by ectopically expressing the wild-type *csrA* allele in the N*csrA.*R6H strain, demonstrating that the motility defect is due to the *csrA* mutation. Because the *csrA* mutant is less motile than the wild type, we conclude that CsrA positively regulates motility in V. cholerae, consistent with the observed downregulation of motility gene expression in the *csrA* mutant.

### CsrA regulates rugose switching in V. cholerae.

In order to cope with extracellular stressors, V. cholerae may alter its outer surface through the excretion of extracellular polysaccharides, resulting in a rugose colony morphology. The rugose morphology arises through phase variation, yielding colonies with a distinctive rough, wrinkled surface (32). The rugose phenotype is associated with an overproduction of biofilms (33) and increased resistance to osmotic and oxidative stressors (33, 34) and antimicrobial compounds (35). The rugose variant has been linked to outbreaks of cholera (35) and has been proposed to confer increased persistence in the environment (36). Many of the regulatory and structural genes known to play a role in the production of the extracellular polysaccharide matrix of V. cholerae (reviewed in reference 37) were identified in the RNA-seq and CsrA-RNA coimmunoprecipitation analyses as being potential targets of CsrA regulation. These include genes encoding the sigma factors RpoS, RpoE, and RpoN, as well as the biofilm-specific transcriptional regulator VpsT (VCA0952), and several genes in the *vpsI*, *vpsII*, and *rbm* biofilm gene clusters (VC0916 to VC0939). While many regulatory genes, including *vpsT*, were expressed at lower levels in the absence of CsrA, a number of the structural genes were differentially regulated through the growth phases, being generally downregulated or neutral in mid-exponential phase but significantly more abundant at stationary phase in the *csrA* mutant. This pattern of expression suggests that CsrA may be required for induction of exopolysaccharide gene expression during rapid growth, but may have a repressive effect in stationary phase, indicating temporal regulation of this process by CsrA. Interestingly, at least one transcript involved in exopolysaccharide production, *rbmA* (VC0928), was identified as an RNA target of CsrA binding (Fig. 3), suggesting direct regulation by CsrA.

**FIG 5:**
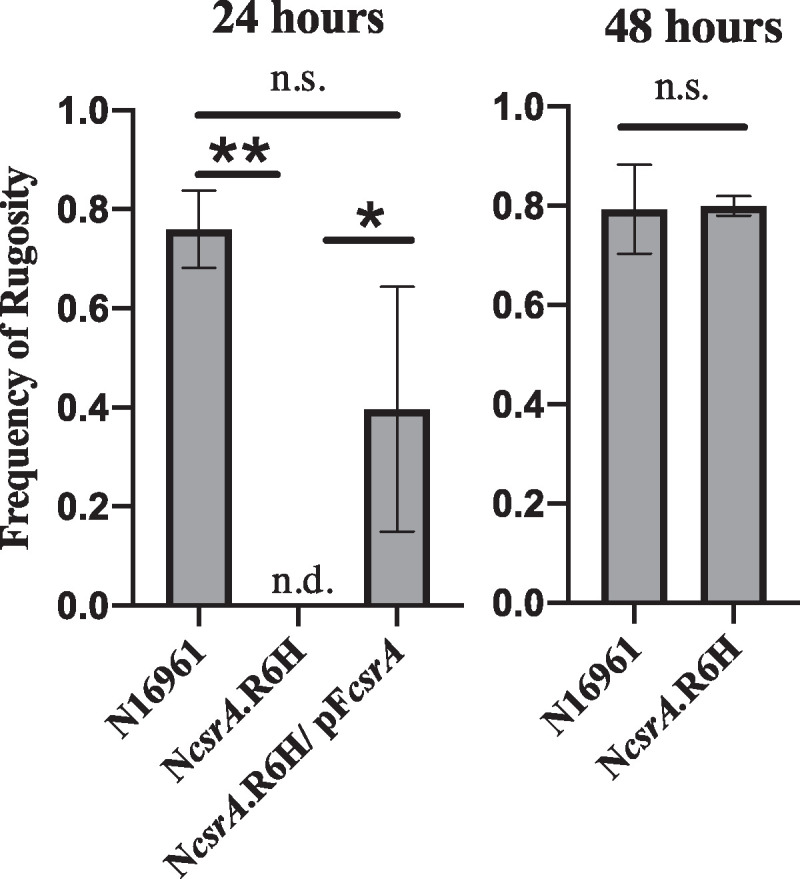
CsrA is required for rugosity at 24 h, but not at 48 h. Cells were plated and the number of rugose colonies for each sample was determined after 24 and 48 h of incubation. The mean of three biological replicates, with two technical replicates for each, is shown for each of the indicated strains, and the error bars are the standard deviation from the mean. A one-way ANOVA, in the Prism 8 software suite, was performed to determine significance (*P* values: *, <0.05; **, <0.01; n.s., not significantly different; n.d., not detected).

To directly test whether CsrA affects the ability of V. cholerae to undergo phase variation and induce exopolysaccharide production, we compared the frequency of transition from the smooth to the rough, or rugose, colony morphology in the wild type and *csrA* mutant at 24 and 48 h of growth without shaking in alkaline peptone water (APW) number 3 growth medium, which promotes switching to the rugose morphology (36, 38). We found that after 24 h of growth, N*csrA*.R6H had not undergone phase variation at all, as none of the cells formed rugose colonies on solid medium. In contrast, 74% of the wild-type colonies displayed the rugose colony morphology at this time point (Fig. 5). However, at 48 h, both the wild-type and the *csrA* mutant colonies displayed the rugose variant phenotype at a frequency of approximately 80%. Additionally, this temporal regulation correlates with the RNA-seq data. Taken together, these observations suggest temporal regulation of rugose switching by CsrA; CsrA may act as a positive regulator of exopolysaccharide production early in the growth cycle, whereas later in the growth cycle the interplay between CsrA and other stationary phase regulators may serve to repress production of the extracellular matrix.

### CsrA binds to the *aphA* mRNA and positively regulates AphA protein production.

**FIG 6:**
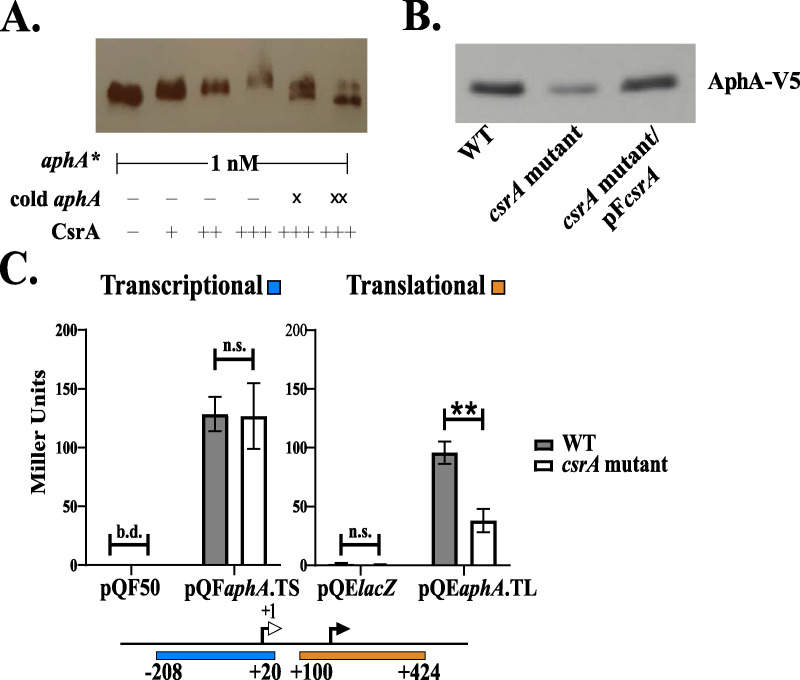
CsrA binds to the *aphA* mRNA and enhances the efficiency of *aphA* translation. (A) The RNA-binding EMSA was performed with purified CsrA (+, 375 nM; ++, 500 nM; +++, 750 nM) (17), biotinylated *aphA* (aphA*), and cold *aphA* (unlabeled *aphA* [X,10 nM; XX, 50 nM]). The *aphA* EMSA is representative of at least three technical replicates. (B) The wild type (*NaphA*-V5), *csrA* mutant (*NcsrA.*R6H.*aphA*-V5), and *csrA* mutant/pF*csrA* (*NcsrA*.R6H.*aphA*-V5/pF*csrA*) were grown in minimal medium supplemented with NRES. Cells were harvested at mid-log phase, and whole-cell proteins were resolved and immunoblotted with anti-V5 antiserum. The immunoblot is representative of at least three biological replicates. (C) The *aphA* transcriptional reporter pQF*aphA*.TS includes the *aphA* promoter sequence, while the *aphA* translational fusion pQE*aphA*.TL includes the 5′UTR and part of the coding sequence. The activity of these reporters was measured in the wild type (WT, N*lacZ*::*kan*) and the *csrA* mutant (N*csrA*.R6H*.lacZ*::*kan*). IPTG (100 μM) was in the growth medium when testing the translational reporter. To generate the plotted means and standard deviations (error bars), three biological replicates were used for both the transcriptional reporter and the translational reporter. The *P* values were calculated using the unpaired, two-tailed Student’s *t* test (*P* value: **, <0.01; b.d., below detection, n.s., not significantly different). Error bars indicate standard deviations.

AphA is a transcriptional regulator essential for the induction of virulence gene expression in V. cholerae (26). AphA also activates biofilm gene expression by inducing expression of *vpsT* (39). We found that the *aphA* (VC2647) transcript was enriched 15-fold in the CsrA-RNA coimmunoprecipitation (Fig. 3), suggesting that *aphA* is a direct regulatory target of CsrA. CsrA plays a key role in both virulence (7) and biofilm (*vps*) gene expression (Table S1), and some of this regulation could be mediated by *aphA*. To confirm that CsrA binds directly to the *aphA* mRNA, we performed an *in vitro* RNA electrophoretic mobility shift assay (EMSA) with purified CsrA and a 3′ biotinylated *aphA* mRNA probe. The *aphA* probe begins at the transcriptional start site, as determined by Papenfort et al. (40), and includes, in addition to the 5′ untranslated region (5′ UTR), 228 nucleotides of the *aphA* coding sequence (+1 to +432 relative to the start of transcription). The sequence of the *aphA* probe contains one GGA motif in the 5′ UTR and four additional GGA motifs in the coding sequence. The RNA EMSA revealed an initial shifted *aphA* species with the addition of 375 nM of purified CsrA (Fig. 6A). As the concentration of CsrA increased, the extent to which the *aphA* species was shifted also increased. To compete with the biotinylated *aphA* mRNA (aphA*), unlabeled *aphA* mRNA was added in 10-fold (lane 5) or 50-fold (lane 6) excess of aphA*. The addition of unlabeled *aphA* competed for CsrA binding, resulting in a reduction in the extent of aphA* species shifting. This result shows that CsrA is capable of binding to the *aphA* mRNA both *in vitro* and *in vivo*, indicating that *aphA* is a direct target of CsrA regulation.

Because both CsrA and AphA are positive regulators of virulence gene expression and *aphA* expression is itself modestly, but positively, influenced by CsrA in exponential growth phase (17) (Fig. S1), we hypothesized that CsrA positively regulates AphA protein production by binding to the *aphA* transcript and increasing the efficiency of translation of the *aphA* mRNA. To determine whether CsrA regulates AphA protein production, we compared the amount of AphA protein produced in the wild type versus the *csrA* mutant. In order to visualize the AphA protein, a V5 epitope tag sequence was fused in-frame to the 3′ end of the *aphA* gene in the chromosome of both the wild type and the *csrA* mutant, giving rise to strains N*aphA*-V5 and N*csrA.*R6H*.aphA*-V5. Strains were grown in defined medium supplemented with NRES, and the level of AphA-V5 was determined by Western blotting using anti-V5 antisera (Fig. 6B). There was less AphA-V5 produced in the *csrA* mutant than in the wild type, and the AphA-V5 level was restored by complementing the *csrA* mutant with the wild type *csrA* allele on a plasmid. This result shows that the observed decrease in AphA-V5 protein production was due to the *csrA* mutation, confirming that CsrA positively regulates AphA protein production.

10.1128/mBio.03380-20.1 FIG S1 CsrA influences *aphA* gene expression during exponential, but not stationary, phase growth. Expression of *aphA* in N*csrA*.R6H compared to N16961 during early exponential, mid-exponential, and stationary phase growth in the RNA-sequencing experiment (Table S1). Download FIG S1, PDF file, 0.1 MB. Copyright © 2021 Butz et al. 2021 Butz et al. This content is distributed under the terms of the Creative Commons Attribution 4.0 International license.

The regulation of AphA protein synthesis by CsrA could occur through an increase in translational efficiency when CsrA binds to the *aphA* transcript. To test whether CsrA affects the translation of *aphA*, a translational fusion of *aphA* to the *lacZ* gene was constructed for testing in the wild type and the *csrA* mutant. For comparison, a transcriptional *aphA*-*lacZ* promoter fusion was generated as well. CsrA is a posttranscriptional regulator with no known direct effects on transcription; however, we wanted to rule out any indirect effects of CsrA on the expression of *aphA* in the reporter assays. In order to ensure all β-galactosidase is produced from the reporter constructs, the strains N*lacz*::kan and N*csrA.lacZ*::kan were used as the wild type and *csrA* mutant (17). The β-galactosidase activity from the transcriptional reporter was the same in both the wild type and the *csrA* mutant (Fig. 6C), consistent with CsrA having no effect on the expression of the *aphA* gene. In contrast, the translational reporter, containing the 5′UTR and partial coding sequence of *aphA* fused to *lacZ*, had significantly reduced β-galactosidase activity in the *csrA* mutant compared with the wild type, suggesting CsrA is required for optimal translation of the *aphA* mRNA. Taken together, these results point to a mechanism of regulation whereby CsrA binds directly to the *aphA* mRNA and enhances its translation. The exact nature of the translational regulation of *aphA* by CsrA remains to be determined.

## DISCUSSION

**FIG 7:**
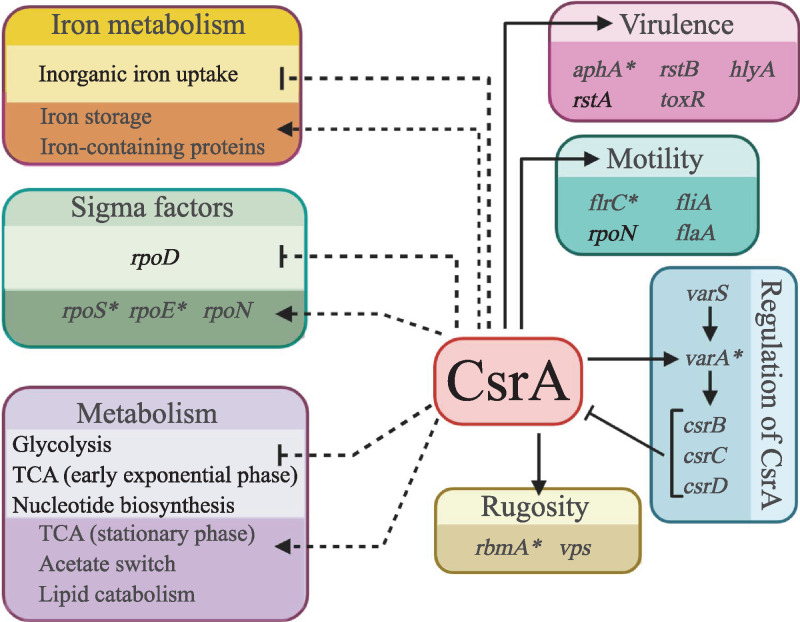
V. cholerae CsrA regulon. Boxes indicate major pathways regulated by CsrA. Solid arrows and bars show processes that have been confirmed by genetic or phenotypic analysis. Asterisks indicate direct targets that have been confirmed by coimmunoprecipitation or EMSA. Figure created with BioRender.com.

In this study, we have begun the characterization of the CsrA regulon in V. cholerae. By comparing differences in gene expression between a *csrA* mutant and its wild-type parental strain at multiple phases of growth, we found that CsrA affects the expression of ∼22% of the V. cholerae transcriptome. Further, by analyzing the effects of CsrA at multiple phases of growth, we were able to identify genes that showed growth-phase-dependent expression patterns, revealing significant changes during the V. cholerae growth cycle that would otherwise have been missed. The regulatory roles of CsrA identified in this analysis are summarized in Fig. 7. It is possible that the number of regulated genes is even greater, because the N*csrA.*R6H mutant strain produces a CsrA protein with partial activity.

The most plausible explanation for the substantial effect of CsrA on the V. cholerae transcriptome is that CsrA regulates other regulators. We found that CsrA directly bound multiple mRNAs that encode transcriptional regulators, two of which are sigma factors, RpoS and RpoE, each controlling a large subset of genes. Regulation of RpoS by CsrA is likely responsible for the large proportion of the genes that were regulated exclusively during stationary phase, and there is substantial overlap between genes regulated by RpoS (31) and CsrA at stationary phase. The importance of CsrA in the adaptation of V. cholerae to the nutrient limitations and other stresses of stationary phase growth was also evident in the categories of genes regulated by CsrA. At stationary phase, CsrA had a repressive effect on genes involved in nucleotide biosynthesis, gene expression, and protein translation, suggesting that CsrA is critical for slowing down metabolism when nutrients are scarce. At the same time, cellular catabolic processes were enriched by CsrA, which would increase the supply of readily metabolizable macromolecules. During stationary phase growth, CsrA repressed genes involved in iron uptake, while increasing expression of genes encoding iron storage and other iron-containing proteins, pointing to a major role for CsrA in both reducing influx of iron and increasing iron storage. By limiting the import of free iron, increasing iron storage, and increasing the expression of iron-binding proteins, CsrA may help to mitigate the toxic effects of free iron in stationary phase (41). Fewer iron-related genes were affected by CsrA in exponential phase, and these included genes encoding iron sulfur cluster-binding proteins. In contrast to the observed regulation in stationary phase, these genes were repressed by CsrA during mid-exponential phase, suggesting that CsrA may serve to redirect the available iron away from iron-intense metabolic pathways and toward essential biosynthetic pathways when iron is less abundant (42). In E. coli, CsrA was found to repress synthesis of intracellular iron storage proteins during growth in exponential phase, thus freeing up iron when the requirement for iron in metabolic and respiratory pathways is high (9, 43). Together with our findings, this suggests that CsrA regulates iron levels to increase access to iron in the rapidly growing cell, but acts to mitigate the toxic effects of free iron when the iron requirements are less.

Central carbon metabolism was also a target of CsrA regulation. This was expected, as CsrA was originally named for its role in carbon storage regulation in E. coli (44). It was shown in E. coli that CsrA activates glycolysis both at mid-exponential phase and stationary phase through positive regulation of the expression and protein production of *pfkA*, which encodes the glycolytic enzyme phosphofructokinase I (9, 45–48). Unlike the findings in E. coli, we did not detect a strong effect of V. cholerae CsrA on glycolysis at mid-exponential phase; the genes within this pathway were mostly unaffected by the *csrA* mutation. CsrA did, however, have an effect on glycolytic gene expression at stationary phase; expression of *pfkA* (VC2689) was significantly more highly expressed in the *csrA* mutant, suggesting that CsrA is a repressor of *pfkA* in V. cholerae, rather than an activator as in E. coli. Additional differences between E. coli and V. cholerae CsrA-mediated regulation of carbon metabolism were observed in the TCA cycle. While E. coli CsrA represses TCA cycle genes under multiple growth conditions (9, 46), V. cholerae CsrA appears to repress expression in early exponential phase, and it clearly activates gene expression in stationary phase. Differences in CsrA-mediated regulation of TCA and glycolysis genes indicate that CsrA can regulate these processes independently in V. cholerae, whereas the regulation of these two pathways appears to be linked in E. coli (45, 46, 49).

Results from the RNA-seq experiments (Table S1) suggest that CsrA may activate the acetate switch in stationary phase. The acetate switch promotes the transition from acetate excretion during rapid growth to acetate assimilation when other, preferred, carbon sources become depleted. Expression of genes involved in the acetate switch, including genes encoding the two-component system CrbRS (VC0303/VC2702), a putative cation-acetate symporter system (VC2704/VC2705), acetyl-CoA synthase (VC0298) (50), and CobB (VC1509), which activates the acetyl-CoA synthase through deacetylation (29), was reduced in the *csrA* mutant at stationary phase, consistent with positive regulation of these genes by CsrA. Although the statistical threshold for inclusion in the data set (*P* < 0.05) was not met in every case for these genes, the overall trend in gene expression suggests that V. cholerae is no longer excreting acetate via glycolysis and mixed acid fermentation in stationary phase, but is instead taking up acetate from the medium and using it in the TCA cycle in a CsrA-dependent process. In addition, the *cobB* (VC1509) transcript was a direct target of CsrA binding at mid-exponential phase (Fig. 3), suggesting CsrA may also regulate acetate metabolism during rapid growth.

We observed that genes within the *de novo* inosine monophosphate (IMP) pathway were significantly enriched in the PANTHER analysis, with 7 out of 12 genes in this category expressed at a higher level in the *csrA* mutant compared to the wild type at stationary phase (Fig. 1B). Activation of the IMP pathway is a crucial step in the stringent response, as it is required for the synthesis of the alarmone (p)ppGpp (51). In E. coli, a *csrA* mutant had an increase in the expression of the (p)ppGpp synthase, *relA*, as well as increased levels of (p)ppGpp, indicating that CsrA acts to repress aspects of the stringent response (52). Interestingly, *relA* (VC2451) was not significantly differentially regulated in the RNA-seq; however, V. cholerae has another (p)ppGpp synthase, *relV* (VC1124) (53), which was upregulated 2.8-fold at stationary phase in N*csrA*.R6H. CsrA-dependent repression of the stringent response in E. coli, together with the overrepresentation of upregulated genes within the *de novo* IMP pathway, and upregulation of *relV* in N*csrA*.R6H, suggest that in the wild type CsrA may repress the stringent response in V. cholerae as well.

In addition to regulation of metabolic pathways, V. cholerae CsrA plays a significant role in control of motility and virulence, and the pattern of CsrA as a regulator of regulators emerges here, as well as in stationary phase regulation. One of the mRNAs enriched in the CsrA-RNA co-IP was VCA0965, which encodes a cyclic di-guanylate (c-di-GMP) synthase. C-di-GMP is a secondary metabolite that has been shown to inversely regulate motility and biofilm formation (30, 54, 55), as well as regulate the expression of V. cholerae virulence genes (56). It has also been shown to regulate the expression of multiple transcriptional regulators such as *vpsT* and *aphA* (57, 58). A decrease in c-di-GMP in the *csrA* mutant could explain the downregulation of *vpsT* expression, as c-di-GMP is a known inducer of *vpsT* expression (34). VpsT is the major activator of genes within the vibrio polysaccharide synthesis (VPS) gene cluster (VC0916 to VC0939), and some of the *vps* genes (VC0931 to VC0938) had reduced expression in the *csrA* mutant at mid-exponential phase (59). This would suggest that CsrA has a positive effect on VPS production in V. cholerae; however, many *vps* genes, including *vpsA* (VC0917) and *rbmA* (VC0928), were significantly upregulated in the *csrA* mutant in stationary phase, indicating the effect of CsrA is to repress these genes in stationary phase. Thus, the effect of CsrA on exopolysaccharide production depends on growth phase, and likely other factors. The timing of expression of different genes involved in exopolysaccharide production could help explain the temporal regulation of rugose switching by CsrA. It is not known if temporal regulation of exopolysaccharide production through CsrA is a general feature of V. cholerae, or whether it is unique to QS-deficient strains.

CsrA positively regulates motility in V. cholerae, as has been described in E. coli. A large proportion of the flagellar assembly genes were downregulated in the *csrA* mutant, and we demonstrated that CsrA coimmunoprecipitated with the transcripts for two regulators of motility genes: (i) *flrC* transcript, encoding the class III transcriptional regulator of motility genes (27), and (ii) *rpoN* mRNA, encoding a sigma factor involved in activating both class II and class III flagellar genes (60). This suggests that CsrA regulates motility at multiple points, and that regulation could be occurring directly, on *flrC*, and both directly and indirectly through *rpoN*. This is of particular interest because it has been demonstrated in V. cholerae that these regulators are also required for proper expression of virulence genes (27). Further, it has been demonstrated that Δ*flaA* or Δ*motAB* mutants, which are nonmotile, do not colonize the infant mouse small intestine, and either fail to express (Δ*flaA*), or misregulate (Δ*motAB*), the *ctxA* gene *in vitro* and *in vivo* (61). Thus, motility and virulence are linked in V. cholerae, and CsrA could affect pathogenesis by regulating motility.

One of the central transcriptional regulators coordinating quorum sensing and other environmental stimuli, such as oxygen levels and pH, with virulence gene expression in V. cholerae is AphA (26). When activated, AphA induces *tcpPH* transcription, and TcpP, together with ToxR, induces *toxT* expression. ToxT, in turn, activates expression of the toxin coregulated pilus, TCP, and cholera toxin, CTX (reviewed in reference 62). The *aphA* transcript was a target of CsrA binding in the CsrA-RNA co-IP, and we demonstrated that CsrA can bind directly to an RNA sequence representing the 5′UTR and initial sequence of the *aphA* mRNA *in vitro*. Validating the relevance of the CsrA-*aphA* interaction, we showed that AphA protein levels were significantly decreased in the *csrA* mutant, and CsrA was required for optimal AphA translation, but not *aphA* transcription. The precise mechanism of enhanced translation by CsrA in V. cholerae is unknown and under investigation.

We previously demonstrated that CsrA is required for colonization of the infant mouse model, and that CsrA positively regulates ToxR protein production. Here, we demonstrate that CsrA positively regulates other processes that are also important for pathogenesis, such as motility and AphA protein production. Virulence gene regulation in V. cholerae comprises an exceedingly complex network of sensory two-component systems, regulatory RNAs and proteins, and small signaling molecules. From our RNA-seq and CsrA-RNA co-IP data, we show that CsrA plays a central role in how signals are sensed and relayed, and in how responses are coordinated. It is likely the colonization defect of the *csrA* mutant is due to effects on several, not just one, pathway leading to virulence gene expression.

## MATERIALS AND METHODS

### Bacterial strains and growth conditions.

All strains (Table S3) were maintained at −80°C in tryptic soy broth plus 20% (vol/vol) glycerol. Cultures were grown at 37°C with shaking in Luria-Bertani (LB) broth (1% [wt/vol] tryptone, 0.5% [wt/vol] yeast extract, and 1% [wt/vol] NaCl) or on LB agar plates. LB overnight cultures inoculated from single colonies were subcultured 1:100 into the minimal medium and grown at 37°C with shaking. The minimal medium, a modified T medium, was supplemented with a mixture of the amino acids asparagine, arginine, glutamate, and serine (NRES), each dissolved in water and added to a final concentration of 3.125 mM. The T medium was modified to contain 0.2% (wt/vol) sucrose, 20 μM FeSO_4_, and VA vitamin solution (https://www.genome.wisc.edu/resources/protocols/ezmedium.htm). Unless stated, all experiments were performed with at least three biological replicates. For the RNA-sequencing experiment, LB cultures from single colonies were grown overnight, then diluted 1:100 in T medium with NRES and grown to early exponential phase (optical density at 650 nM [OD_650_] ∼0.1), mid-exponential phase (OD_650_ ∼0.5), and stationary phase (OD_650_ ∼2.0). For the CsrA-RNA coimmunoprecipitation experiment, LB cultures from single colonies were grown overnight, then diluted 1:100 in T medium with NRES and grown to mid-exponential phase (OD_650_ ∼0.5). The following antibiotics were used at the indicated concentrations: for E. coli, 50 μg/ml kanamycin, 50 μg/ml ampicillin, 30 μg/ml chloramphenicol, and 250 μg/ml carbenicillin; for V. cholerae, 50 μg/ml kanamycin, 25 μg/ml ampicillin, 6 μg/ml chloramphenicol, 125 μg/ml carbenicillin, and 20 μg/ml polymyxin B.

10.1128/mBio.03380-20.4 TABLE S3 Strains and plasmids. Download TABLE S3, DOCX file, 0.02 MB. Copyright © 2021 Butz et al. 2021 Butz et al. This content is distributed under the terms of the Creative Commons Attribution 4.0 International license.

### Isolation of V. cholerae RNA.

Approximately 10^9^ bacterial cells, grown in minimal medium supplemented with NRES, were pelleted by centrifugation and resuspended in 100 μl of the growth medium. One ml of RNA-BEE (Tel-Test, Inc., Friendswood, TX), a solution containing phenol and quinidine thiocyanate, was added to the cells. Two hundred microliters of RNase-free chloroform was added, and the mixture was centrifuged at 4°C to separate the organic and aquatic phases. The aquatic phase was removed by pipetting and added to an equal volume of RNase-free isopropanol, and placed at −80°C overnight. The precipitated nucleic acids were pelleted by centrifuging the samples at 21,100 × *g* for 10 min at 4°C. The nucleic acids were washed with 500 μl of 75% RNase-free ethanol. The dried RNA/DNA pellet was resuspended in 20 to 50 μl of RNase-free H_2_O. The nucleic acids were treated with DNase I (New England BioLabs [NEB], Ipswich, MA) as per the manufacturer’s directions and subsequently stored at −80°C overnight. The RNA was washed with 500 μl of 75% RNase-free ethanol and the dried RNA pellet was resuspended in 20 to 50 μl of RNase-free H_2_O. The isolated RNA was either used immediately or placed at −80°C until further use.

### Whole-genome RNA-sequencing.

The purified RNA was sent to the Genomic Sequencing and Analysis Facility at The University of Texas at Austin, where the RNA samples were assembled into a single-ended RNA-seq library and sequenced by Illumina-based next-generation sequencing. The RNA-seq samples were sequenced on the HiSeq 4000, resulting in an average of 26.9 million reads per sample. The Illumina reads were then imported into the CLC Genomics Workbench 11.0.1 (Qiagen, Valencia, CA) and mapped to the N16961 reference genome (NC_002505 and NC_002506), with a slight modification. The experimentally determined 5′ UTRs were added into the gene track to permit the RNA reads to properly align to the 5′ UTR regions within the genome (40). The standard CLC Genomic Workbench parameters were then used to map each sample’s reads individually to the genome. The expression value for each gene was reported as a transcripts per million (TPM). The TPM expression value was selected because it normalizes the number of reads for a gene to the length of that gene. The samples were normalized by quantile, grouped, and comparisons between the *csrA* mutant and the wild type were made. The Baggerley’s test was used to determine statistical significance (63). From this analysis, a gene was considered to be significantly differentially expressed between N16961 and N*csrA.*R6H if that gene had a false discovery rate (FDR) *P* value of <0.05.

### GO ontology analysis.

Genes with a statistically significant (FDR *P* value of <0.05) and a weighted proportions fold change of >2-fold were selected for analysis via PANTHER Gene Ontology (GO). The statistical overrepresentation test with the GO biological process complete annotation data set was used. To determine processes that were overrepresented, Fisher’s exact test was applied, and categories with an FDR *P* value of <0.05 were considered significant.

### CsrA-RNA coimmunoprecipitation.

A *csrA* allele encoding a CsrA protein tagged with a single V5 tag sequence on the C terminus, inserted into the inducible vector pBAD18-cm, was generously provided by Bryan Davies (The University of Texas at Austin). The N*csrA*.R6H strain harboring an arabinose-inducible V5 epitope-tagged *csrA* allele was grown to mid-exponential phase and *csrA*-V5 expression was induced. RNA was harvested from 5% of the lysate and the remainder was incubated with magnetic beads coated with anti-V5 antibody. The beads were collected and washed, and then CsrA-bound RNA was isolated from the beads. CsrA-V5 expression was induced in mid-exponential phase culture by adding 0.1% arabinose to the medium for 15 min. The cells were centrifuged and resuspended in 2.5 ml of binding/wash buffer (100 mM MOPS [pH 7.0] [KOH], 10 mM MgCl_2_, 100 mM KCl). The cells were lysed in a French pressure cell with a final volume of ∼2 ml. One hundred microliters of the cell lysate was added directly to RNA-Bee, and the RNA was further purified. The remaining lysate was incubated with protein G Dynabeads (Thermo Fisher Scientific, Waltham, MA) coated with anti-V5 antibodies (Sigma-Aldrich, St. Louis, MO; number V8012) for an hour. The beads were washed 4 times with binding/wash buffer, resuspended in 100 μl of buffer, and added to RNA-Bee for further RNA purification.

### Generating cDNA and quantitative PCR.

SuperScript III (Thermo Fisher Scientific) was used to reverse transcribe 2 μg of RNA into cDNA using the random primers supplied with the SuperScript III kit. Fresh cDNA was diluted 1:10 with ddH_2_O and was used immediately. Primers were designed by using Primer3 (http://bioinfo.ut.ee/primer3-0.4.0/) and were selected to be located within the first half of the target gene, and to produce a product between 50 and 150 nucleotides in length. Real-time qPCR was performed with Power SYBR Green (Thermo Fisher Scientific). The Applied Biosystems ViiA 7 instrument was used with the following parameters for qPCR: (i) holding stage, 50°C for 2 min and 95°C for 10 min; (ii) PCR stage, 95°C for 15 sec and 60°C for 1 min, repeated 40 times with fluorescence recorded at 60°C; and (iii) melting curve stage, 90°C for 15 sec, 60°C for 1 min, then 95°C for 15 sec with the fluorescence recorded every 0.05 sec. Each reaction produced only one melting curve, indicating only one target was amplified during the qPCR. The threshold cycle (ΔΔ*C_T_*) method was used to determine the relative amount of RNA present in the sample tested. The *atpI* gene was used as an internal reference to normalize the CsrA-RNA coimmunoprecipitation because we have previously shown that CsrA does not bind to the mRNA transcript of this gene (17).

### Frequency of rugosity.

To determine the percentage of rugose conversion, the strains were grown in alkaline peptone water (APW) number 3 medium, which has been previously shown to promote high rates of conversion (36). APW number 3 (3 ml) was inoculated with a single colony and placed at 37°C without shaking. At 24 or 48 h, the cultures were vigorously pipetted up and down through a P1000, then a P100 Gilson pipetman tip, to break up the cellular aggregates. The cells were serially diluted in saline, and the appropriate dilution to obtain 20 to 40 colonies was plated onto LB plates without antibiotics and incubated for 24 h at 37°C. After an additional 24 to 48 h of growth at room temperature, the number of smooth and rugose colonies was enumerated. To calculate the frequency of conversion, the number of rugose colonies was divided by the total number of colonies on the plate. At least 100 colonies were observed for each strain.

### Motility.

A 0.1 to 10-μl pipette tip was used to stab a single colony into the motility agar. The motility medium was T medium supplemented with NRES, and contained 0.3% agar. The inoculated plates were placed upside-down at 37°C for 24 h. Relative rates of motility were then determined by measuring the diameter of growth.

### Construction of V. cholerae strains and plasmids.

All strains and plasmids constructed during this study are listed in Table S3. Constructing the *aphA-V5* chromosomal fusion proteins was performed by allelic exchange as previously described (17). The primers used to generate the suicide vector pS*aphA*-V5 are listed in Table S4. To create the *aphA* transcriptional fusion, an AT-nucleotide-rich (65% AT) region directly upstream of the *aphA* coding sequence was selected as the likely promoter. This region is 228 nucleotides in length (2821121 to 2821348) and goes from −208 to +20 relative to the transcriptional start site determined by Papenfort et al. (40). The predicted promoter region was PCR amplified using primers listed in Table S4. This PCR fragment was digested with NcoI and BamHI and cloned into the NcoI and BamHI sites located upstream of a promoterless *lacZ* gene in the plasmid pQF50, generating the full-length reporter construct pQF*aphA*.TS. The translational reporter pQE*lacZ* (17) was used to generate the *aphA* translational reporter. The *aphA* translational reporter construct begins at +100 and goes to +424 relative to the start of transcription (2821428 to 2821752). The primers used to generate this fragment are listed in Table S4. The promoterless *aphA* construct was cloned into the *Mfe*I and NotI sites downstream of the T5 promoter in pQELacZ to generate the translational reporter plasmid pQE*aphA*.TL.

10.1128/mBio.03380-20.5 TABLE S4 Primers. Download TABLE S4, DOCX file, 0.01 MB. Copyright © 2021 Butz et al. 2021 Butz et al. This content is distributed under the terms of the Creative Commons Attribution 4.0 International license.

### SDS-PAGE and immunoblotting.

Cultures were grown to mid-exponential phase (OD_650_ ∼0.5) and samples containing an equivalent number of cells were resuspended in Laemmli SDS sample buffer (64). Samples were resolved by electrophoresis through 10% SDS-polyacrylamide gels and the proteins were visualized by Coomassie brilliant blue staining or immunoblotting. The proteins for immunoblotting were transferred to a 0.45-μm-pore-size nitrocellulose membrane (GE Healthcare, Chicago, IL). The AphA-V5 fusion protein was immunodetected by mouse monoclonal anti-V5 antibodies (diluted 1:10,000) (Sigma-Aldrich, number V8012). To visualize the protein, horseradish peroxidase (HRP)-conjugated goat anti-mouse IgG (diluted 1:10,000) (Bio-Rad Laboratories, Hercules, CA) was used. To ensure equal loading, the relative densities of the immunoblotted samples were assessed by Coomassie staining of a duplicate gel.

### Generating RNA probes.

The MEGAshortscript kit (Thermo Fisher Scientific) was used to *in vitro* transcribe RNA. For this method, the PCR product of the *aphA* mRNA was generated using a forward primer containing the T7 promoter (Table S4). The region PCR amplified includes 204 nucleotides upstream from the ATG and 228 nucleotides into the coding region (2821329 to 2821760). The purified T7 PCR product was used as the template at a concentration of between 25 and 125 nM. After the *in vitro* transcription reaction, the RNA in the 20-μl reaction was precipitated by adding 5 μl RNase-free 3 M sodium acetate, followed by 125 μl of 100% RNase-free isopropanol. Once the precipitated RNA pelleted dried completely, the RNA was resuspended in RNase-free H_2_O. The Pierce RNA 3′ end biotinylation kit (Thermo Fisher Scientific) was then used as per the manufacturer’s directions to biotinylate the RNA. The biotinylated RNA was precipitated and washed, as described above, and resuspended in RNase-free H_2_O. The biotinylated RNA was either used immediately or placed at −80°C until further use.

### RNA electrophoretic mobility shift assays.

RNA oligonucleotides were made as described above. CsrA was purified as described in reference 17. The CsrA-RNA electrophoretic mobility shift assay (EMSA) samples were prepared according to the LightShift Chemiluminescent RNA EMSA kit (Thermo Fisher Scientific) directions, with the slight modification of a 10-μl final volume instead of a 25-μl final volume. CsrA-RNA complexes were resolved by electrophoresis through a 5% nondenaturing polyacrylamide gel, transferred to the BrightStar-Plus (Thermo Fisher Scientific) positively charged nylon membrane, and UV cross-linked with 150 mJ. The LightShift Chemiluminescent RNA EMSA kit was used to visualize the biotinylated RNA.

### *lacZ*-based reporter assays.

Strains harboring either a transcriptional or translational *lacZ*-based reporter were grown to mid-exponential phase in minimal medium with or without NRES, and the optical density at OD_650_ was recorded. To ensure equivalent expression from the translational reporter, 100 μM IPTG (isopropyl-β-D-thiogalactopyranoside) was added to the growth medium. Cell suspension (100 μl) was placed into 900 μl of Z-Buffer (60 mM Na_2_HPO_4_, 40 mM NaH_2_PO_4_, 10 mM KCl, 1 mM MgSO_4_, and 2.5 mM β-ME) (65). The cells were permeabilized by adding 20 μl of chloroform, and then 10 μl of 0.1% SDS. After 15 min, 200 μl of freshly made ONPG solution (4 mg/ml ONPG dissolved in phosphate buffer [60 mM Na_2_HPO_4_, 40 mM NaH_2_PO_4_]) was added to the cell suspension in Z-Buffer solution. The duration of time between adding the ONPG and the solution turning light yellow was recorded, and once color change was observed, 500 μl of stop solution (1 M Na_2_CO_3_) was added. The OD_420_ and OD_550_ were recorded to determine the β-galactosidase activity. Then, by using the following calculation, the Miller units were determined:

(1) $$\text{Miller units }=\;\frac{1000(OD_{420}\;-\;\left(1.75*OD_{550}\right))}{\mathrm{time}(\min)*\mathrm{Vol}\left(\mathrm{mL}\right)*OD_{650}}$$
