# Supplementary material for: Regulatory Effects of CsrA in Vibrio cholerae
Source: mBio. 2021 Feb 2;12(1):e03380-20. doi: 10.1128/mBio.03380-20 (PMC7858070; doi:10.1128/mBio.03380-20)
Supplement: FIG S1 [file mBio.03380-20-sf001.pdf]

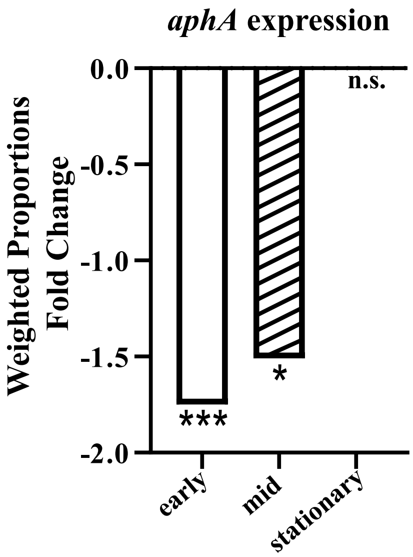

Figure S1. CsrA influences *aphA* gene expression during exponential, but not stationary, phase growth. Expression of *aphA* in N*csrA*.R6H compared to N16961 during early exponential, mid-exponential, and stationary phase growth in the RNA-sequencing experiment (Table S1).
