# Supplementary material for: Regulatory Effects of CsrA in Vibrio cholerae
Source: mBio. 2021 Feb 2;12(1):e03380-20. doi: 10.1128/mBio.03380-20 (PMC7858070; doi:10.1128/mBio.03380-20)
Supplement: TABLE S3 [file mBio.03380-20-st003.docx]

Table S3. Strains and plasmids

| **Strains** | **Description** | **Source or reference** |
| --- | --- | --- |
| ***E. coli*** | | |
| DH5α *(λpir)* | *Escherichia coli* cloning strain | J. Kaper |
| Sm10 *(λpir)* | *Escherichia coli* cloning and mobilization strain for pCVD442N | (66) |
| MM294/pRK2013 | *Escherichia coli* conjugation helper strain | R. Meyer |
| **V. cholerae** | | |
| N16961 | Wild-type *V. cholerae* El Tor biotype | R. A. Finkelstein |
| N*csrA*.R6H | N16961 *csrA* with an Arg to His mutation at position 6 | (7) |
| N*lacZ::kan* | N16961 *lacZ*::kan | (17) |
| N*csrA.*R6H*.lacZ::kan* | N*csrA*.*R6H* *lacZ*::kan | (17) |
| N*aphA-*V5 | N16961 containing the *aohA*-V5 allele | This study |
| N*csrA.*R6H*.aphA*-V5 | N*csrA*.*R6H* containing the *aphA*-V5 allele | This study |
| **Plasmids** |  |  |
| pCC1 | Single-copy no. cloning vector; cam^R^ | Epicenter |
| pCVD442N | Suicide vector pG704 carrying *sacB*; amp^R^, Suc^s^ | (67) |
| pQF50 | Promoterless *lacZ* reporter plasmid; amp^R^ | (68) |
| pQE*lacZ* | pQE2 carrying a *lacZ* allele with the first 9 amino acids not included; amp^R^ | (17) |
| pF*csrA* | pCC1 carrying *csrA*; cam^R^ | (7) |
| pBAD*csrA-*V5 | pBAD18-cm carrying *csrA*-V5 with a 2 glycine linker; cam^R^ | Bryan Davies |
| pS*aphA*-V5 | pCVD442N carrying a construct to chromosomally V5 epitope tag the C-terminus of AphA with a 2 glycine linker; amp^R^ | This study |
| pQFaphA.TS | pQF50 carrying the *aphA* promoter; amp^R^ | This study |
| pQE*aphA*.TL | pQELacZ carrying the *aphA* translational fusion to lacZ; amp^R^ | This study |
