# Supplementary material for: Regulatory Effects of CsrA in Vibrio cholerae
Source: mBio. 2021 Feb 2;12(1):e03380-20. doi: 10.1128/mBio.03380-20 (PMC7858070; doi:10.1128/mBio.03380-20)
Supplement: TABLE S4 [file mBio.03380-20-st004.docx]

Table S4. Primers

| **Purpose** | | **Primer name** | **5' to 3'** |
| --- | --- | --- | --- |
| **Fusion protein primers** | | | |
| V5-tagging AphA | AphA.F | | GGTGAACCAGACTTTGTTCCTTACTTCCG |
|  | AphA-V5.R | | GGTGAACCAGACTTTGTTCCTTACTTCCG |
|  | AphA-V5.F | | ATCCCTAACCCTCTCCTCGGTCTCGATTCTACGTAAGCCAAGCCAAACCTGTCG |
|  | AphA.R | | ATCCCTAACCCTCTCCTCGGTCTCGATTCTACGTAAGCCAAGCCAAACCTGTCG |
| **Reporter Primers** | | | |
| Transcriptional reporter | | AphA.ts.F | ATGCCCATGGCCTTGATGGTGACCATTGCG |
|  |  | AphA.ts.R | GTCAGGATCCTTACATTACTTATCACAGAGGG |
| Translational reporter | | AphA.tl.F | ATGCCAATTGAATTTTGCGCTGCAGGTATTTAAATGC |
|  |  | AphA.tl.R | ATGCGCGGCCGCCTTGGGTTATAGAATAAACCTTGCG |
| **RNA-EMSA primers** | | | |
| AphA EMSA oligo | | T7.AphA.F | CGCGCATTAATACGACTCACTATAGGGCTCTGTGATAAGTAATGTAAAGC |
|  |  | T7.AphA.R | GCGCTGCGGCCAGCTTGGGTTATAGAATAAACCTTGCG |
| **qPCR** | | | |
| qPCR | | CsrB.F | GGACATTGAACGGACGCAATCG |
|  |  | CsrB.R | TGATAGTACGCTGCAATCCG |
|  |  | aphA.F | AATGGGGGAACAGGGTTTAG |
|  |  | aphA.R | AAACCTTGCGATCTGGTTTG |
|  |  | flrC.F | CGGACATTTCCGTGAAGACT |
|  |  | flrC.R | TTTTGCAGTGACGCTCAATC |
|  |  | rpoS.F | GCACCATTCGTCTACCGATT |
|  |  | rpoS.R | TGGTGTAGGTTCGTGGTCAA |
|  |  | rpoE.F | TAGCGCAGGAAGCGTTTATT |
|  |  | rpoE.R | TAGCGCAGGAAGCGTTTATT |
|  |  | nrpII.F | CGGCATCACAGGTATGACAG |
|  |  | nrpII.R | CGCGGTACAGTTCAGTGTGT |
|  |  | espC.F | AAGATGGTGTGGGTCGTCTC |
|  |  | espC.R | CGGCTCAGTAAACACACCAA |
|  |  | VC1604.F | AGACACTGCAACAAGCGATG |
|  |  | VC1604.R | CAAAATCCGCCAACTGTTTT |
|  |  | cobB.F | AAAGAGTTGCAGGGCAGTGT |
|  |  | cob.R | ACCGTTTGATTGGACTCAGG |
|  |  | rbmA.F | CAGCCAGTGATTGAAGCAAA |
|  |  | rbmA.R | ATCCACCGTTACTTGCCAAC |
|  |  | VCA0965.F | CTTATGCGCTGGCCTTAGTC |
|  |  | VCA0965.R | TCGCTGCACTGTAAACCAAG |
|  |  | atpI.F | CTTAGTCGGTGGTGGCATTT |
|  |  | atpI.R | CTGCAAACAGGAAAGCACAA |
